# Effect of Unsaturated Sn Atoms on Gas-Sensing Property in Hydrogenated SnO_2_ Nanocrystals and Sensing Mechanism

**DOI:** 10.1038/s41598-017-00891-5

**Published:** 2017-04-27

**Authors:** Y. Yuan, Y. Wang, M. Wang, J. Liu, C. Pei, B. Liu, H. Zhao, S. Liu, H. Yang

**Affiliations:** 1 Shaanxi Key Laboratory for Advanced Energy Devices; Shaanxi Engineering Laboratory for Advanced Energy Technology; Key Laboratory of Macromolecular Science of Shaanxi Province, School of Materials Science and Engineering, Shaanxi Normal University, Xi’an, 710119 China; 2 Key Laboratory of Applied Surface and Colloid Chemistry, National Ministry of Education; Shaanxi Key Laboratory for Advanced Energy Devices; Shaanxi Engineering Lab for Advanced Energy Technology, School of Materials Science and Engineering, Shaanxi Normal University, Xi’an, 710119 China

## Abstract

Sensing reaction mechanism is crucial for enhancing the sensing performance of semiconductor-based sensing materials. Here we show a new strategy to enhancing sensing performance of SnO_2_ nanocrystals by increasing the density of unsaturated Sn atoms with dangling bonds at the SnO_2_ surface through hydrogenation. A concept of the surface unsaturated Sn atoms serving as active sites for the sensing reaction is proposed, and the sensing mechanism is described in detail at atomic and molecule level for the first time. Sensing properties of other metal oxide sensors and catalytic activity of other catalysts may be improved by using the hydrogenation strategy. The concept of the surface unsaturated metal atoms serving as active sites may be very useful for understanding the sensing and catalytic reaction mechanisms and designing advanced sensing sensors, catalysts and photoelectronic devices.

## Introduction

As an important n-type metal-oxide semiconductor with a band gap of 3.6 eV at 300 K^1^, tin dioxide (SnO_2_) is well known for its numerous potential applications in field emission^2–4^, lithium ion batteries^5–7^, photocatalysis^8, 9^, dye-sensitizd solar cells^10, 11^, perovskite solar cells^12–15^, supercapacitors^16, 17^, light emitting devices^18^ and so on. On the other hand, SnO_2_ is considered to be one of the best known gas-sensing materials due to its remarkable receptivity variation in gaseous environment and excellent chemical stability^19–22^. Over the past decades, with the development of nanoscience and nanotechnology, SnO_2_ nanomaterials with controlled morphologies, including nanoring^20^, nanowires^23, 24^, nanobelts^25^, nanotubes^26, 27^, nanosheets^28^, hollow spheres^29, 30^, flower-like structure^31, 32^, nanopolyhedrons^33^, hierarchical nanoarchitectures^34, 35^, octahedra^21, 22^ and porous nanospheres^36^ have been employed to fabricate gas sensors for detection of inflammable and toxic gases and volatile organic compounds (VOC) to improve gas-sensing properties. It was found that sensitivity of the sensors based SnO_2_ nanostructured materials is increased by exposing high-energy facets^21, 22^ and increasing surface area^26, 31, 36^. Additionally, the sensitivity and selectivity of SnO_2_-based sensors can be significantly improved through doping with Cu, and Zn elements^37, 38^, decorating with Ag_2_O^39^, PdO^40^, Ag^41^ and Pd^42^ nanoparticles, and forming SnO_2_-ZnO heteronanostructures^43^.

Metal-oxide gas sensors like SnO_2_ operate on the basis of the change of the electrical resistance upon exposure to air or a test gas^44^. The variations of the resistances are brought about by the oxidation-reduction reaction of the adsorbed oxygen with the test gas taking place on the metal-oxide surface^45, 46^. For this reason, the sensing behaviors of metal oxides should be very sensitive to the surface adsorption oxygen ability of the sensing materials. The adsorption oxygen ability may be enhanced by increasing density of the unsaturated metallic atoms with dangling bonds on the surface of metal-oxide sensors through hydrogenation. However, such a strategy for increasing sensing properties has never been reported up to now.

Herein, we demonstrated the enhanced volatile-organic-compound sensors based on the hydrogenated SnO_2_ nanocrystals for the first time. The hydrogenated SnO_2_ nanocrystals displayed far higher response towards ethanol, methanol and triethylamine than SnO_2_ samples without hydrogenation, and the gas-sensing sensitivity was further increased with the hydrogenation time. The excellent gas-sensing performance arises from the increased density of the unsaturated Sn atoms with dangling bonds through hydrogenation, a concept of the unsaturated Sn atom serving as an active site for the sensing reaction is thus proposed, and a new sensing reaction mechanism is described in detail.

## Results

**Figure 1:**
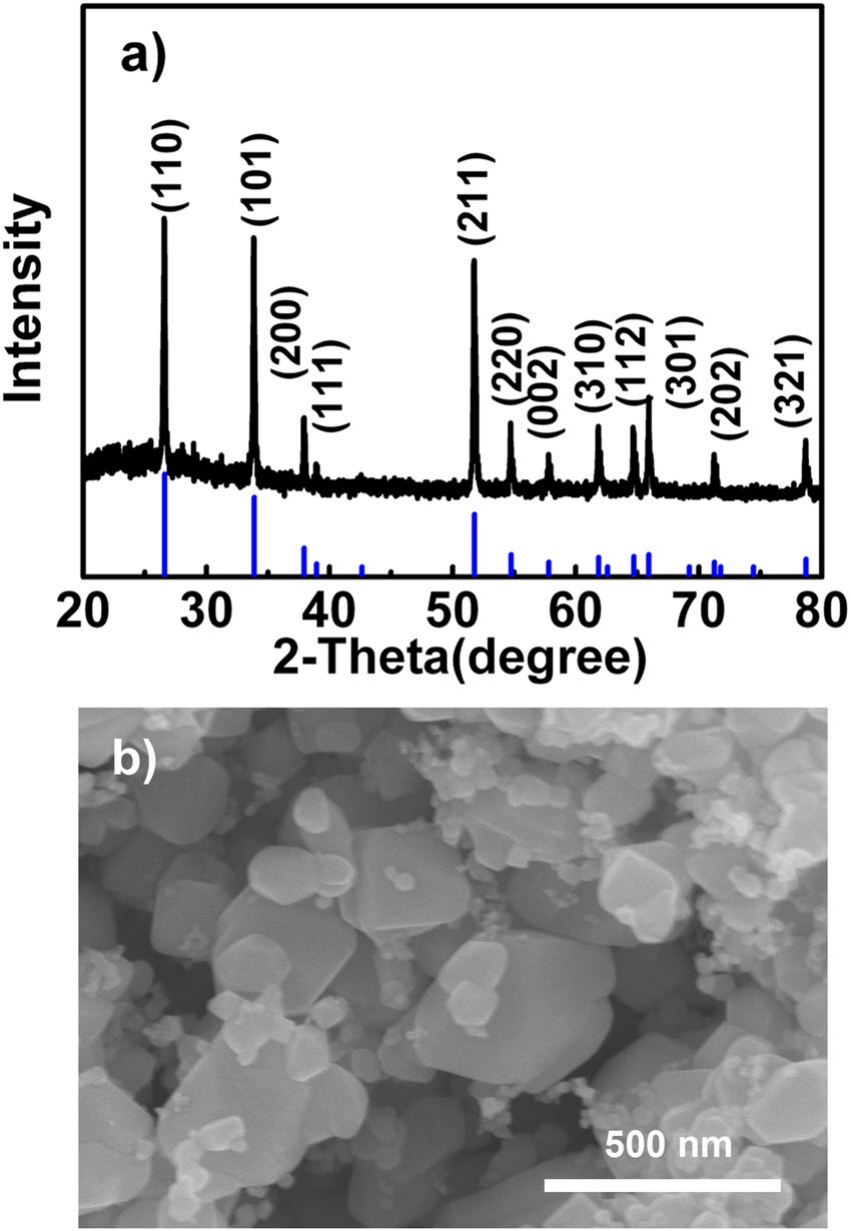
XRD and SEM characterizations of the SnO_2_ samples without hydrogenation. (**a**) XRD pattern, (**b**) SEM image.

Morphology and crystal structure of SnO_2_ samples. Figure 1a shows X-ray diffraction (XRD) pattern of the SnO_2_ samples without hydrogenation. In the XRD pattern, all diffraction peaks were attributed to the pure tetragonal phase with cell constants of a = b = 4.738 Å and c = 3.187 Å (Joint Committee on Powder Diffraction Standards No. 41-1445). Scanning electron microscope (SEM) image shown in Fig. 1b indicated that the SnO_2_ samples without hydrogenation consists of nanocrystals with irregular morphology and the sizes of 50–500 nm. The SnO_2_ nanocrystals were hydrogenated for 5, 10 and 15 h at 150 °C, and the as-obtained sample was labeled SnO_2_-H-5, SnO_2_-H-10 and SnO_2_-H-15, respectively. The three kinds of hydrogenated samples were characterized with FESEM and XRD, and the results are shown in Supporting Information Fig. 1. It can be seen that after H_2_ reduction the morphology and crystal structure of SnO_2_ nanocrystals remain unchanged. The as-obtained hydrogenated samples still consist of rutile SnO_2_ nanocrystals with various sizes.

**Figure 2:**
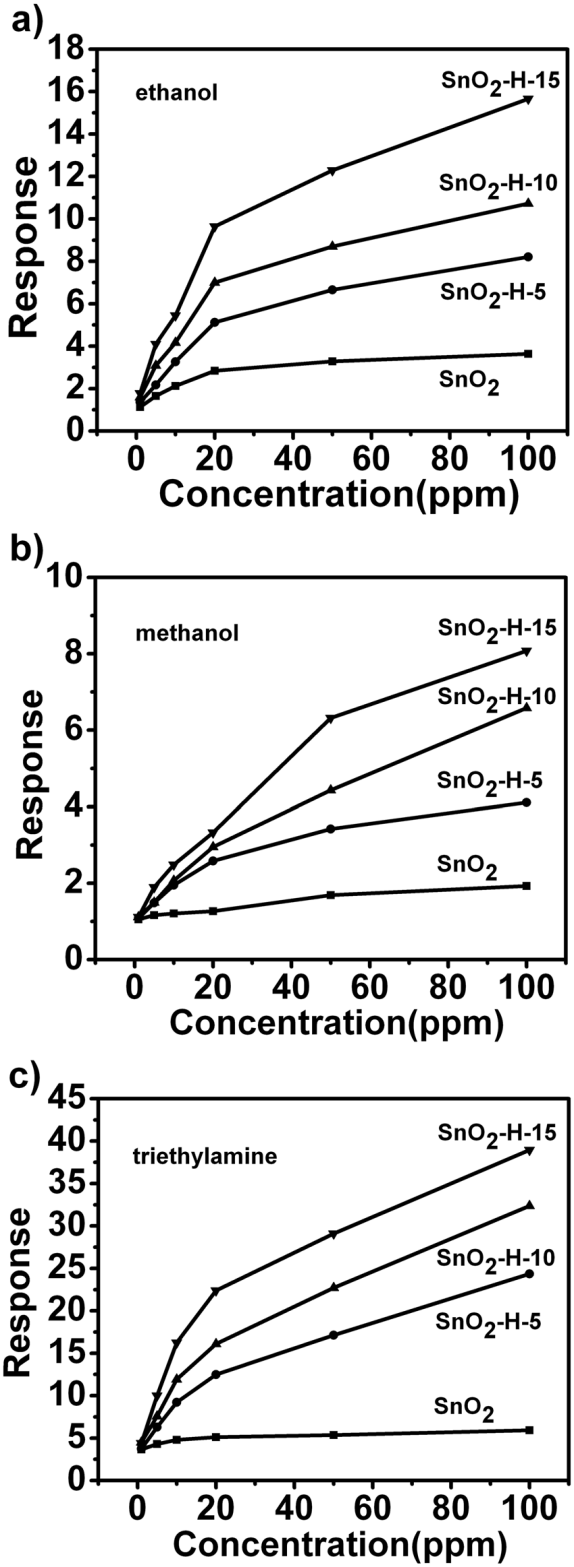
The response curves of the sensors based on the hydrogenated and non-hydrogenated SnO_2_ nanocrystals to different concentrations of VOCs with 50% of relative humidity at 350 °C.

Gas-sensing Performance. The transient response characteristics of the hydrogenated and non-hydrogenated SnO_2_ nanocrystals to different concentrations of ethanol, methanol or triethylamine are displayed in Supporting Information Fig. 2. When the VOC was injected, the electric resistance of four types of SnO_2_ nanocrystal sensors decreased suddenly, and then increased rapidly and recovered to their respective initial resistance after release of the VOC vapor. The resistance change of four kinds of SnO_2_ nanocrystal sensors is in accordance with the typical sensing property of the n-type semiconductor^32^. Figure 2a–c shows sensing response curves of the three types of SnO_2_ nanocrystal sensors to ethanol, methanol and triethylamine of different concentration, respectively. It can be clearly seen that the hydrogenated SnO_2_ nanocrystal sensors have higher response than the nanocrystals without hydrogenation for three kinds of VOC vapors. The response value of SnO_2_ sensors increases further with prolonging the hydrogenation time.

Moreover, to investigate stability of the sensor based on the SnO_2_-H-15, after the first measurement the hydrogenated SnO_2_ sensor was stored in air and kept working at 350 °C for subsequent sensing property tests. After the sensor fabrication and aging for 7 days, a series of tests were conducted with 100 ppm of ethanol. The result was shown in Supporting Information Fig. 3. It was found that response value of the hydrogenated SnO_2_ sensor to 100 ppm of ethanol is between 18.9 and 20.5 during the test of 31 days, revealing that the hydrogenated SnO_2_ sensor demonstrated good long-term stability.

**Figure 3:**
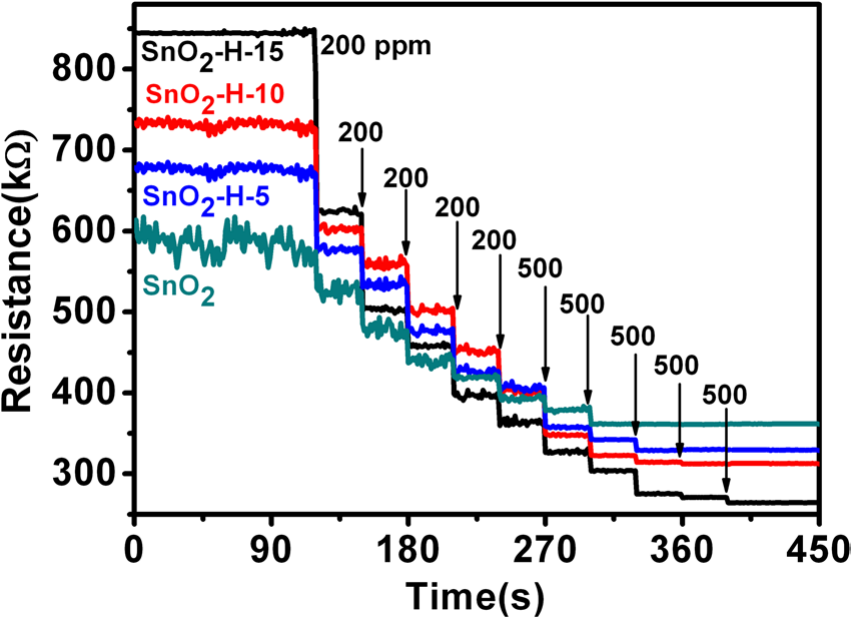
The resistance of the sensors based on the hydrogenated and non-hydrogenated SnO_2_ nanocrystals in air and in different concentrations of ethanol vapors with of 50% relative humidity at 350 °C. The concentration labeled in the figure is the ethanol concentration of injection each time.

## Discussion

It is well accepted that the resistance change of the metal oxide semiconductor based sensors like SnO_2_ is based on the exchange of charges between the absorbed gaseous species and the surface of metal oxide sensing materials^20–22, 44, 47, 48^. In order to understand the role of hydrogenation in the increase of response of SnO_2_ nanocrystal sensor towards VOC, resistances of the four types of SnO_2_ nanocrystal sensors in air and in ethanol vapors of different concentrations were measured, and the results are displayed in Fig. 3. It was found that the hydrogenated SnO_2_ nanocrystal sensors have higher resistance values than SnO_2_ samples without hydrogenation, and the resistance change follows the order of SnO_2_-H-15 > SnO_2_-H-10 > SnO_2_-H-5 > SnO_2_. The resistance values of the four types of SnO_2_ sensors reduce with the injection of ethanol, and finally reach a constant value. The saturated ethanol concentrations for SnO_2_, SnO_2_-H-5, SnO_2_-H-10 and SnO_2_-H-15 are 2000, 2500, 3000 and 3500 ppm, respectively (Table 1). In the saturated ethanol vapor environment, the hydrogenated SnO_2_ nanocrystal sensors have lower electric resistances than the sensors based on the SnO_2_ samples without hydrogenation, and the resistance follows the order of SnO_2_ > SnO_2_-H-5 > SnO_2_-H-10 > SnO_2_-H-15.

**Table 1:** Resistances in air (R_a_) and in the saturated ethanol (R_sg_) and the ethanol saturated concentrations of the hydrogenated and non-hydrogenated SnO_2_ samples.

Samples	R_a_ (kΩ)	R_sg_ (kΩ)	The saturated concentration of ethanol (ppm)
SnO_2_	585.24	361.82	2000
SnO_2_-H-5	675.98	329.30	2500
SnO_2_-H-10	731.35	264.60	3000
SnO_2_-H-15	844.30	244.52	3500

**Figure 4:**
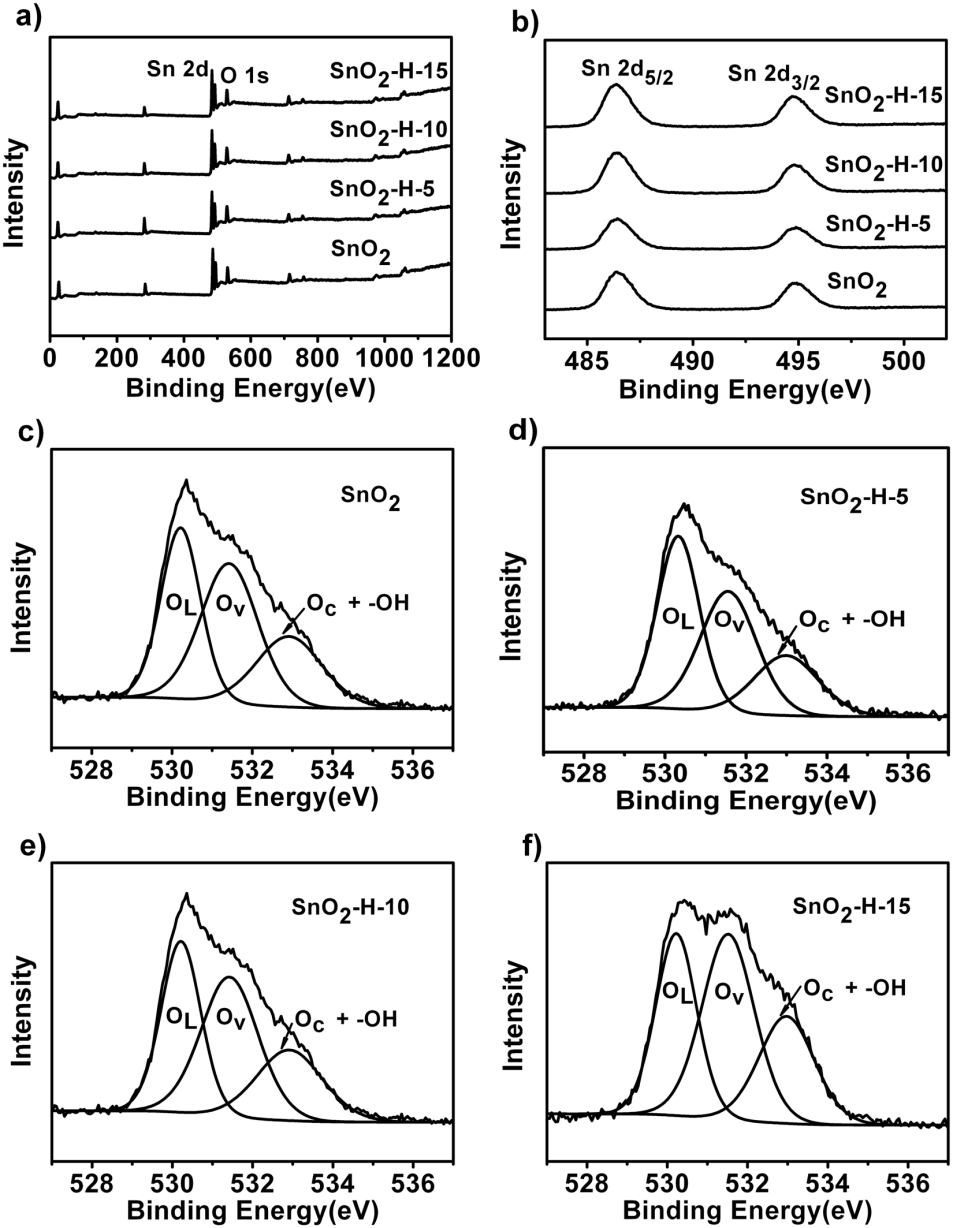
XPS Characterizations of the hydrogenated and non-hydrogenated SnO_2_ nanocrystals. (**a**) The survey spectra. (**b**) Sn 2d5/2, 2d3/2 and (**c**–**f**) O 1 s spectra of (**c**) SnO_2_, (**d**) SnO_2_-H-5, (**e**) SnO_2_-H-10 and (**f**) SnO_2_-H-15.

**Figure 5:**
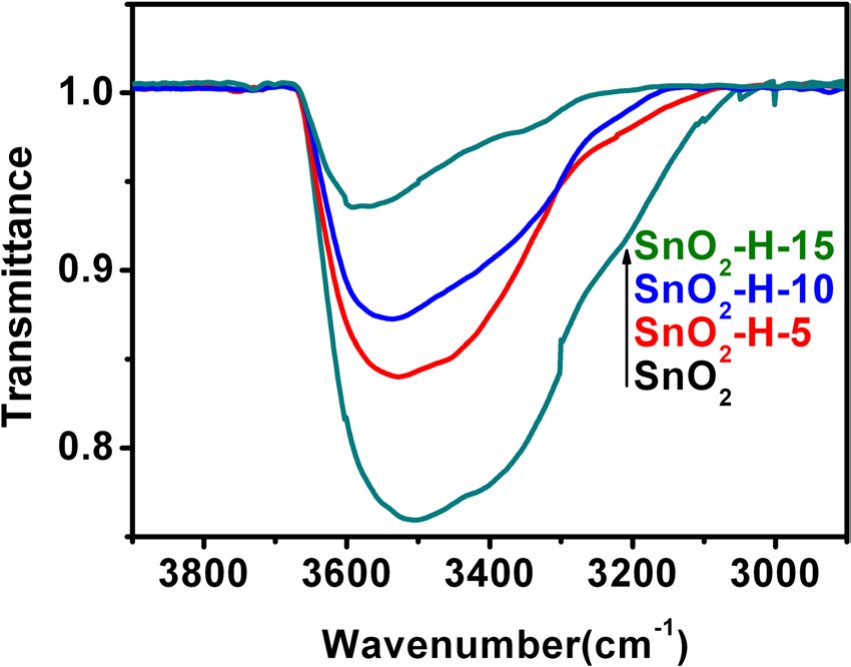
IR spectra of the hydrogenated and non-hydrogenated SnO_2_ nanocrystals.

To determine the state of oxygen species on the surface of the hydrogenated and non-hydrogenated SnO_2_ samples, X-ray photoelectron spectroscopy (XPS) analysis was carried out. Figure 4a–b shows the survey spectra, Sn 2p_5/2_ and 2p_3/2_ spectra, respectively. The binding energy of Sn 2p_5/2_ and 2p_3/2_ is identified at 486.12 and 494.52 eV, respectively (Fig. 4b). Figure 4c–f shows the O 1s spectra from the four kinds of SnO_2_ samples. It was clearly observed that all O 1s XPS peaks can be decomposed into three Gaussian components centered at about 529, 531, and 533 eV, respectively. The three components are indexed to O^2−^ ions in SnO_2_ lattice (O_L_), O^2−^ ions in oxygen-deficient regions (Ov) and chemisorbed oxygen (O_C_) species and –OH groups, respectively^46, 49^. Intensities of O_V_ and O_C_ from the SnO_2_-H-5, SnO_2_-H-10 and SnO_2_-H-15 are higher than those from SnO_2_ samples without hydrogenation. The relative percentages of the O_L_ and O_V_ components as well as O_C_ and –OH mixed components from the SnO_2_-H-5, SnO_2_-H-10, SnO_2_-H-15 and SnO_2_ are summarized in Supplementary Table 1. It is apparent that the relative percentages of the O_V_ components as well as O_C_ and –OH mixed components of the SnO_2_ nanocrystals can be increased through hydrogenation. Additionally, IR spectra of the four kinds of SnO_2_ nanocrystal samples were investigated and the results are shown in Fig. 5. The band at about 3500 cm^−1^ is attributed to the asymmetrical stretching vibration of O-H group. Apparently, the hydrogenated SnO_2_ nanocrystals had lower intensities of the O-H vibration model than SnO_2_ samples without hydrogenation, and the O-H vibration intensity reduced further with increasing hydrogenation time. Therefore, the hydrogenated SnO_2_ samples have higher relative percentage of O_C_ components compared with SnO_2_ samples without hydrogenation, and the relative percentage of O_C_ components further increases with the hydrogenation time. On basis of the experimental results, we concluded that the enhanced sensing property of the hydrogenated SnO_2_ VOC sensors may derive from the increase on the relative percentages of the O_V_ and O_C_ components and the decrease in the amount of O-H groups.

**Figure 6:**
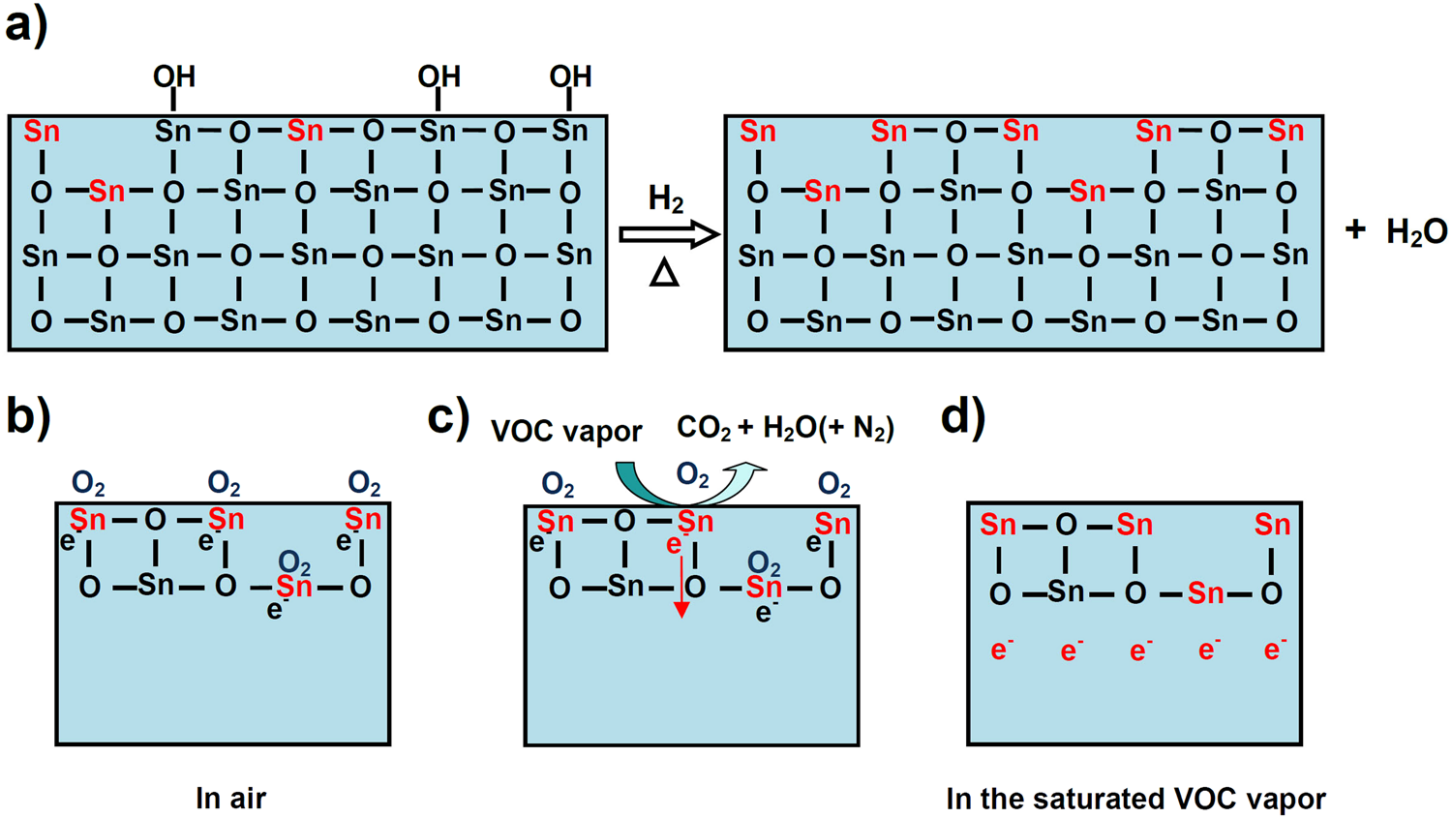
Schematic diagrams of sensing mechanism. (**a**) Hydrogenation reaction of SnO_2_ nanocrystals. (**b–d**) Sensing reaction mechanism of the hydrogenated SnO_2_ nanocrystals at atomic and molecule level. The black e− and red e− represent the absorbed electrons by O2 and free electrons, respectively.

When SnO_2_ nanocrystals were hydrogenated, the O-H groups and O ions at the surface reacted with H_2_ to form H_2_O, and thus more the unsaturated Sn atoms with dangling bonds were formed at the SnO_2_ surface, as a result, density of unsaturated Sn atoms with dangling bonds increases at the surface, as shown in Fig. 6a. Therefore, we considered that the unsaturated Sn atoms with dangling bonds at the surface may play a pivotal role in the enhancement of gas-sensing property. The surface unsaturated Sn atoms with dangling bonds may serve as an active site for the sensing reaction.

As we know, SnO_2_ is a typical n-type semiconductor sensing material with oxygen vacancies, in which electrons participate in the electric conduction process^50, 51^. Like ZnO^52^, TiO_2_^53^ and Fe_2_O_3_^54^, the loss of oxygen in SnO_2_ create non-contributing (extra) electrons. As shown in Fig. 6a, the presence of oxygen vacancies necessarily led to the production of the unsaturated Sn atoms with dangling bonds, and thus we consider that the Sn atoms with dangling bonds can provide free electrons. In air, the unsaturated Sn atoms with dangling bonds at the surface of SnO_2_ sensing material have reducing capacity and adsorb oxygen molecules due to the deficiency of oxygen. The adsorbed oxygen molecules have good oxidation capacity and can draw free electrons in SnO_2_ sensing material, the electrons captured by the adsorbed oxygen can not participate in the electric conduction process. As a result, the number of free electrons within SnO_2_ decreases, and the SnO_2_ sensing material thus shows a high resistance state, as shown in Fig. 6b. The density of unsaturated Sn atoms with dangling bonds increase at the SnO_2_ surface is increased through hydrogenation (Fig. 6a), and thus the amounts of the adsorbed oxygen and the electrons captured by the adsorbed oxygen are enhanced. Therefore, in comparison with SnO_2_ samples without hydrogenation, the hydrogenated SnO_2_ samples have less free electrons and show higher resistance. When the SnO_2_ sensor is exposed to a VOC vapor, the VOC gas molecules are oxidized into CO_2_ and H_2_O (H_2_O + N_2_) by surface-adsorbed oxygen molecules, and thus the adsorbed oxygen was removed^47, 48^. The electrons captured by the adsorbed oxygen molecules are released into SnO_2_, the number of free electrons in SnO_2_ increases, and thus resistance value reduces, as shown in Fig. 6c. In the saturated VOC vapor, all the adsorbed oxygen molecules are removed, all the electrons captured by the adsorbed oxygen became into free electrons, and thus the electric resistance value is constant and the smallest, as shown in Fig. 6d. A total of electron in SnO_2_ sensing materials can be increased with an increase on the density of unsaturated Sn atoms with dangling bonds at the SnO_2_ surface because that the Sn atoms with dangling bonds can provide extra electrons. Therefore, in the saturated ethanol, the hydrogenated SnO_2_ nanocrystals with higher densities of unsaturated Sn atoms with dangling bonds have more free electrons and lower electric resistances than SnO_2_ samples without hydrogenation. Moreover, based on the experimental results in Fig. 3, we considered that the unsaturated Sn atoms with dangling bonds at SnO_2_ surface can catalyze the reaction of the chemisorbed oxygen with the VOC molecules. The hydrogenated SnO_2_ nanocrystal sensors have higher density of sensing reaction active sites (the unsaturated Sn atoms with dangling bonds) than SnO_2_ samples without hydrogenation, and thus demonstrate higher response towards the VOC vapors.

In summary, the hydrogenated SnO_2_ nanocrystals exhibit superior gas-sensing performance, compared with SnO_2_ samples without hydrogenation. The enhanced sensing performances originate from the increased density of the unsaturated Sn atoms with dangling bonds at the SnO_2_ surface through hydrogenation. The unsaturated Sn atoms with dangling bonds are regarded as active sites of the sensing reaction, and the sensing mechanism is firstly elaborated at atomic and molecule level. The hydrogenation may be a general strategy for improving sensing performances of metal oxide sensors and catalytic activities of catalysts. The concept of the unsaturated metal atoms with dangling bonds serving as the reaction active sites not only can deepen understanding of the sensing and catalytic reaction mechanisms, but also provides now insights into the design and fabrication of highly efficient sensing materials, catalysts and photoelectronic devices.

## Methods

### Preparation of samples

SnO_2_ nanocrystals were purchased from Sinopharm Chemical Reagent Co., Ltd. (Shanghai, China). SnO_2_ nanocrystals were hydrogenated: 100 mg of SnO_2_ nanocrystals were heated in a horizontal furnace and maintained at 150 °C for 5, 10 or 15 h under a H_2_ gas flow to obtain hydrogenated SnO_2_ nanocrystals.

### Characterization of SnO_2_ samples

The crystal structure of the hydrogenated and non-hydrogenated SnO_2_ nanocrystals were characterized by X-ray diffraction (Haoyuan DX-2700, Dandong, China) using Cu Kα1 radiation with 2θ ranging from 20° to 80°. The morphology was analyzed by field-emission scanning electron microscope (Hitachi SU8020, Japan) with an acceleration voltage of 20 kV. The surface compositions were determined on an X-ray photoelectron spectroscope (Kratos Axis ultra, Japan) and on an infrared (IR) spectrometer (Bruker Tensor 27, Germany) by mixing 0.001 g of sample with 0.100 g of KBr and pressing into tablet.

### Gas-sensing measurements

Measurements on gas sensitivity of SnO_2_ samples were performed using a WS-30A system (Weisheng Instruments Co., Zhengzhou, China). In a typical test, a sensor was fabricated by coating a certain amount of SnO_2_ paste (consisting of SnO_2_ nanocrystals and the terpineol solvent) onto a ceramic tube with Au electrodes and Pt conducting wires. A Ni-Cr filament was inserted in the tube as a heater element to provide the operation temperature from 200 to 400 °C. To improve the device’s stability, the as-prepared SnO_2_ sensors were aged at 350 °C for 7 days before testing. Measurement of gas-sensing property has been described in the reference^32^. The response of the SnO_2_ sensor is defined as the ratio R_a_/R_g_, where R_a_ and R_g_ are the resistances of the sensor in air and in the test gas at the operation temperature of about 350 °C, respectively.

### Data availability

The data that support the findings of this study are available from the corresponding author upon request.

## Electronic supplementary material

Supplemental materials
