# Supplementary material for: Effect of Unsaturated Sn Atoms on Gas-Sensing Property in Hydrogenated SnO2 Nanocrystals and Sensing Mechanism
Source: Sci Rep. 2017 Apr 27;7:1231. doi: 10.1038/s41598-017-00891-5 (PMC5430629; doi:10.1038/s41598-017-00891-5)
Supplement: Supplementary file 1 — Supplemental materials [file 41598_2017_891_MOESM1_ESM.pdf]

Supporting Information

Title: Effect of Unsaturated Sn Atoms on Gas-Sensing Property in Hydrogenated SnO_2 Nanocrystals and Sensing Mechanism

Yukun Yuan[1], Ye Wang[1], Miao Wang[1], Junfang Liu[1], Cuijin Pei[1], Bin Liu[1], Hua Zhao[1], Shengzhong (Frank) Liu[2] and Heqing Yang[1]*

Y. Yuan, Y. Wang, M. Wang , J. Liu, C. Pei, B. Liu, H. Zhao, Prof. S. Liu, Prof. H. Yang

[1]Shaanxi Key Laboratory for Advanced Energy Devices; Shaanxi Engineering Laboratory for Advanced Energy Technology; Key Laboratory of Macromolecular Science of Shaanxi Province, School of Materials Science and Engineering, Shaanxi Normal University,
Xi'an, 710119, China
E-mail:hqyang@snnu.edu.cn

Prof. Shengzhong (Frank) Liu
[2]Key Laboratory of Applied Surface and Colloid Chemistry, National Ministry of Education; Shaanxi Key Laboratory for Advanced Energy Devices; Shaanxi Engineering Lab for Advanced Energy Technology, School of Materials Science and Engineering, Shaanxi Normal University,
Xi'an 710119, China

Supplementary Figure 1

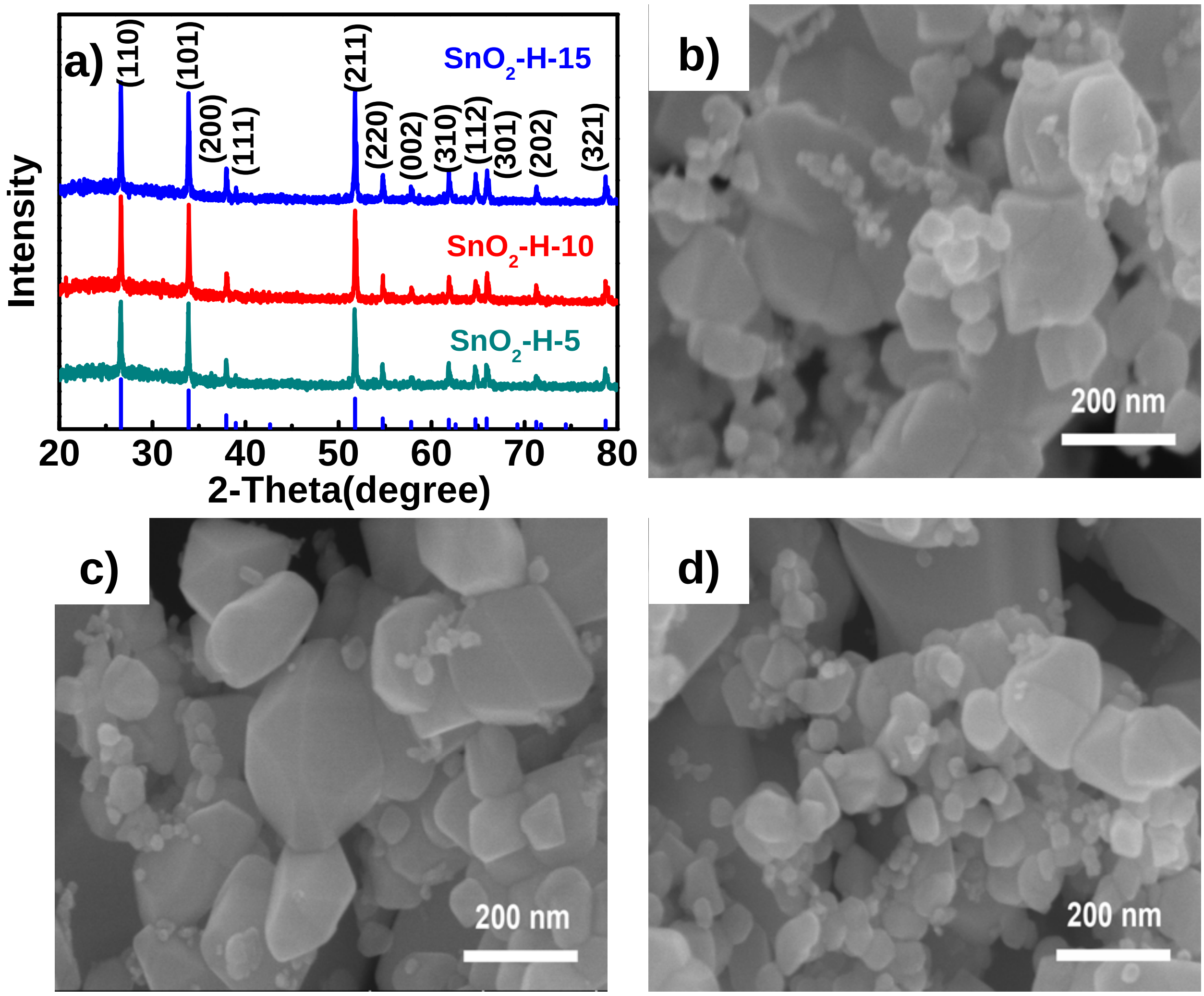

Supplementary Figure 1 | (a) XRD patterns and **(b-d)** FESEM images of the SnO_2-H-5, SnO_2-H-10 and SnO_2-H-15.

Supplementary Figure 2

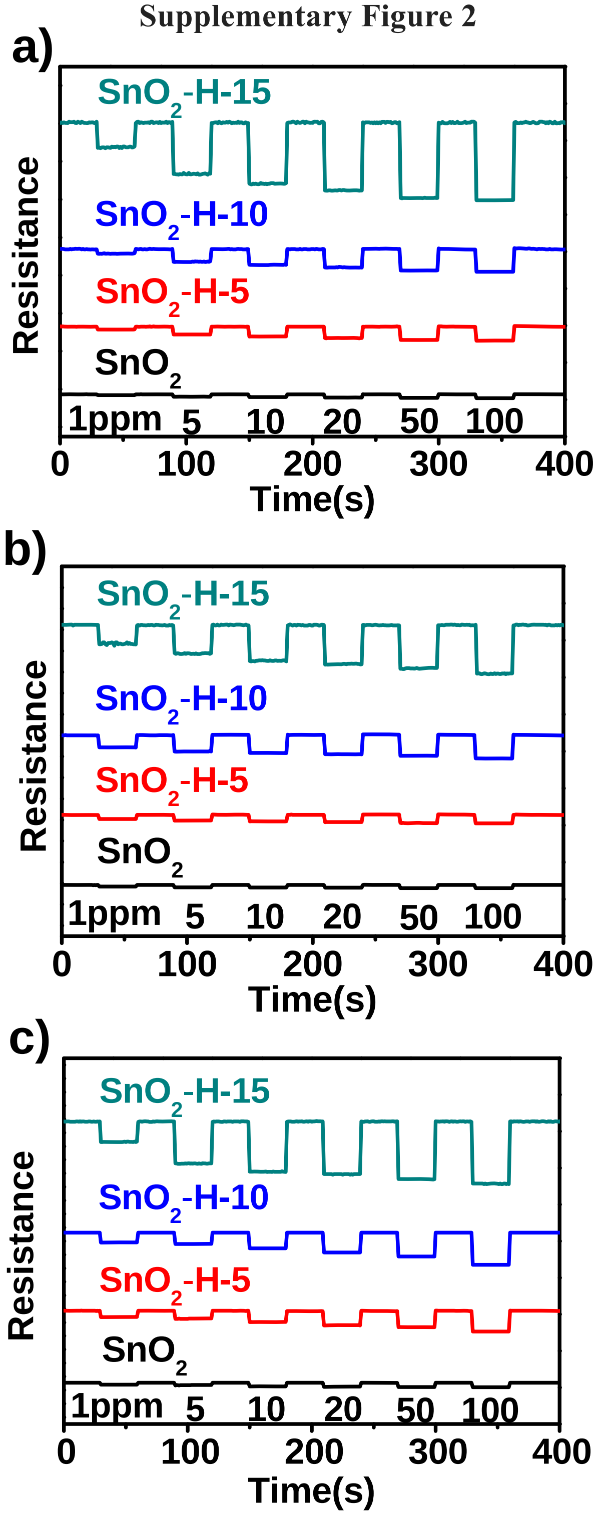

Supplementary Figure 2 | **Transient response and recovery curves of the sensors based on the hydrogenated and non-hydrogenated SnO_2 samples to different concentrations of (a) ethanol, (b) methanol and (c) triethylamin with 50% of relative humidity at 350°C.**

Supplementary Figure 3

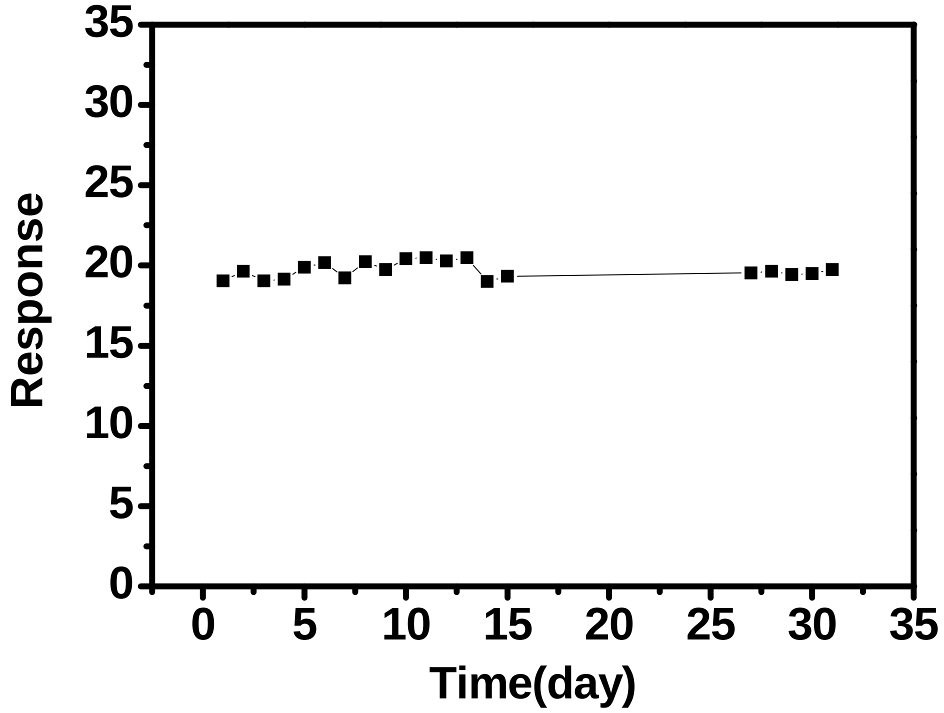

Supplementary Figure 3 | Long-term stability of the sensor based on the SnO_2-H-15 sample to 100 ppm ethanol with 3%-35% of relative humidity at 350◦C.

Supplementary Table 1

Supplementary Table 1 | Fitting result of O 1s XPS spectra of the hydrogenated and non-hydrogenated SnO_2 samples.

SnO_2 sample		O_L (Sn-O)	O_V (vacancy)	O_C (chemisorbed) and -OH
SnO_2	binding energy (eV)	530.27	531.44	532.85
	relative percentage (%)	41.59	37.79	20.61
SnO_2-H-5	binding energy (eV)	530.33	531.56	533.00
	relative percentage (%)	40.52	37.98	21.50
SnO_2-H-10	binding energy (eV)	530.22	531.42	532.92
	relative percentage (%)	34.85	41.20	23.95
SnO_2-H-15	binding energy (eV)	530.22	531.52	532.97
	relative percentage (%)	32.15	42.14	25.71
